# Direct microhaplotype genotyping for GT-seq (Genotyping-in-Thousands by Sequencing) using a diploid abundance model

**DOI:** 10.1371/journal.pcbi.1014808

**Published:** 2026-09-18

**Authors:** Nathan R. Campbell, Amanda R. Campbell, Shannon K. Blair, Amanda J. Finger

**Affiliations:** 1 GTseek LLC, Twin Falls, Idaho, United States of America; 2 University of California – Davis, Davis, California, United States of America; University of Helsinki: Helsingin Yliopisto, FINLAND

## Abstract

GT-seq (Genotyping-in-Thousands by Sequencing) is widely used for high-throughput amplicon genotyping, but most analytical pipelines focus on single SNPs or rely on alignment-based variant calling. Here we present a direct microhaplotype genotyping framework that leverages the high read depth and low error rates typical of paired-end Illumina and Element sequencing. The pipeline first identifies primer-bounded reads and resolves paired-end sequences into quality-aware consensus amplicon sequences. Within each sample and locus, unique sequences are ranked by read abundance and the top one or two sequences are retained as directly observed haplotypes. These alleles are aggregated across samples to construct a catalog of observed haplotypes for each locus. In a second pass, reads are assigned to catalog haplotypes by exact sequence matching to produce diploid genotypes. Finally, catalog haplotype sequences are compared to identify phased SNP and collapsed indel variation. Optionally, catalog haplotypes may be aligned to a reference genome to project observed variants onto genomic coordinates and generate standards-compliant VCF output. This framework enables robust, microhaplotype genotyping directly from high-depth amplicon sequencing data. Comparison with an independent BWA/BCFtools alignment-based workflow demonstrated 99.67% genotype concordance across 102,520 genotype comparisons spanning 1,085 SNPs in 96 individuals. Genotype concordance remained above 99.4% even at the minimum supported sequencing depth of 10 reads per locus, demonstrating robust performance across a broad range of sequencing depths.

## Author summary

GT-seq has become a widely adopted method for genotyping hundreds of genetic markers across thousands of individuals because it combines low cost with high throughput. Existing analytical workflows commonly genotype predefined SNP or haplotype targets within each amplicon rather than reconstructing the complete amplicon sequence before identifying polymorphisms. We developed an open-source analysis framework that instead reconstructs complete amplicon haplotypes directly from paired-end sequencing data before identifying individual polymorphisms. This haplotype-first approach generates phased multi-allelic microhaplotypes without requiring conventional alignment-based variant calling while remaining compatible with standard VCF-based workflows. Using GT-seq data from a 410-locus panel, we demonstrate greater than 99.6% genotype concordance with an independent alignment-based pipeline and show that treating GT-seq amplicons as microhaplotypes increases mean marker information content by approximately 42% compared with single-SNP analysis. Because the method operates entirely in software, many existing GT-seq panels and datasets can be reanalyzed immediately as microhaplotypes without changes to laboratory protocols, providing a practical way to increase the value of previously generated sequencing data.

## Introduction

Targeted amplicon sequencing has become a widely adopted approach for high-throughput genotyping in ecological, conservation, fisheries, aquaculture, and breeding studies. Among these methods, Genotyping-in-Thousands by sequencing (GT-seq) has emerged as an efficient and cost-effective strategy for simultaneously genotyping hundreds of loci across thousands of individuals using highly multiplexed PCR amplification followed by next-generation sequencing [1]. Since its introduction, GT-seq panels have been developed for a broad range of species and applications, including population structure, mixed-stock analysis, hybridization, and long-term genetic monitoring of natural populations [2,3]. Because GT-seq targets a defined set of informative loci rather than requiring genome-wide sequencing, it provides a scalable and economical approach for studies requiring large sample sizes. One of its most important applications is parentage and kinship analysis, where high-throughput SNP panels have been used to assign parentage, reconstruct pedigrees, estimate relatedness, and inform breeding and conservation management decisions [2,4]. The ability to genotype thousands of individuals at relatively low cost makes GT-seq particularly well suited for kinship-based studies in species with large populations or complex breeding systems, where both analytical throughput and marker information content are critical for accurate relationship inference.

Most analytical approaches developed for GT-seq data genotype predetermined variants within targeted amplicons, including approaches that identify allele-specific sequences using primer and internal probe sequences [1,5]. While effective, these approaches begin with predefined variant targets rather than reconstructing complete amplicon haplotypes directly from sequencing reads. Because each amplicon typically spans a short genomic region, multiple polymorphic sites may occur within the same amplicon and can be observed simultaneously on individual sequencing reads. These combinations of linked polymorphisms form haplotypes that may contain substantially more information than individual SNP markers.

Conventional analytical workflows infer haplotypes from previously identified SNP genotypes, either through statistical phasing or by combining variants observed after reference alignment. In contrast, GT-seq amplicons frequently encompass complete microhaplotype loci, allowing the underlying haplotypes to be observed directly within individual sequencing reads. This observation suggests an alternative analytical strategy in which complete haplotypes are identified first and individual polymorphisms are subsequently extracted from the reconstructed haplotypes.

Microhaplotypes, defined as short genomic regions containing two or more closely spaced polymorphisms observed on the same DNA fragment, have emerged as powerful genetic markers for applications including ancestry inference, individual identification, and kinship analysis [6,7]. Because multiple SNPs are observed together on the same sequencing read, microhaplotypes produce multi-allelic markers with higher heterozygosity and greater discriminatory power than single SNP loci. Indeed, several studies have demonstrated that microhaplotypes can substantially improve the statistical power of relatedness analyses. Because each locus may produce multiple haplotype alleles rather than two SNP states, microhaplotypes increase the effective information content of genetic markers and improve the ability to distinguish closely related individuals [6–8].

Targeted amplicon sequencing approaches such as GT-seq are therefore particularly well suited for microhaplotype genotyping. Paired-end sequencing reads frequently span the entire amplified region, allowing complete haplotypes to be observed directly rather than inferred statistically from independent SNP calls. Furthermore, the high sequencing depth typical of GT-seq experiments provides a strong signal for distinguishing true allele sequences from sequencing errors based on read abundance.

Here we describe a direct microhaplotype genotyping framework for GT-seq amplicon sequencing data that reverses the conventional order of variant analysis. Rather than first identifying SNPs and subsequently reconstructing haplotypes, the pipeline first reconstructs complete primer-bounded amplicon sequences and calls diploid haplotypes directly from observed sequence abundance. Observed alleles from all samples are then aggregated to construct a catalog of unique haplotypes for each locus. In a second pass, sequencing reads are assigned to catalog haplotypes by exact sequence matching to produce diploid genotype calls. Polymorphic sites are subsequently extracted from the catalog haplotypes to generate phased microhaplotype representations, and catalog haplotypes may optionally be aligned to a reference genome to project SNP and indel variants onto genomic coordinates and generate standards-compliant VCF output. Because many existing GT-seq panels already span multiple polymorphic sites, this framework enables direct microhaplotype genotyping without modification to established laboratory protocols.

## Materials and methods

### Ethics statement

All fish sampling and handling were conducted by the Fish Conservation and Culture Laboratory (FCCL) in accordance with institutional and governmental guidelines and were approved by the University of California-Davis Institutional Animal Care and Use Committee under IACUC protocol number 23041.

### Analysis pipeline overview

**Fig 1:**
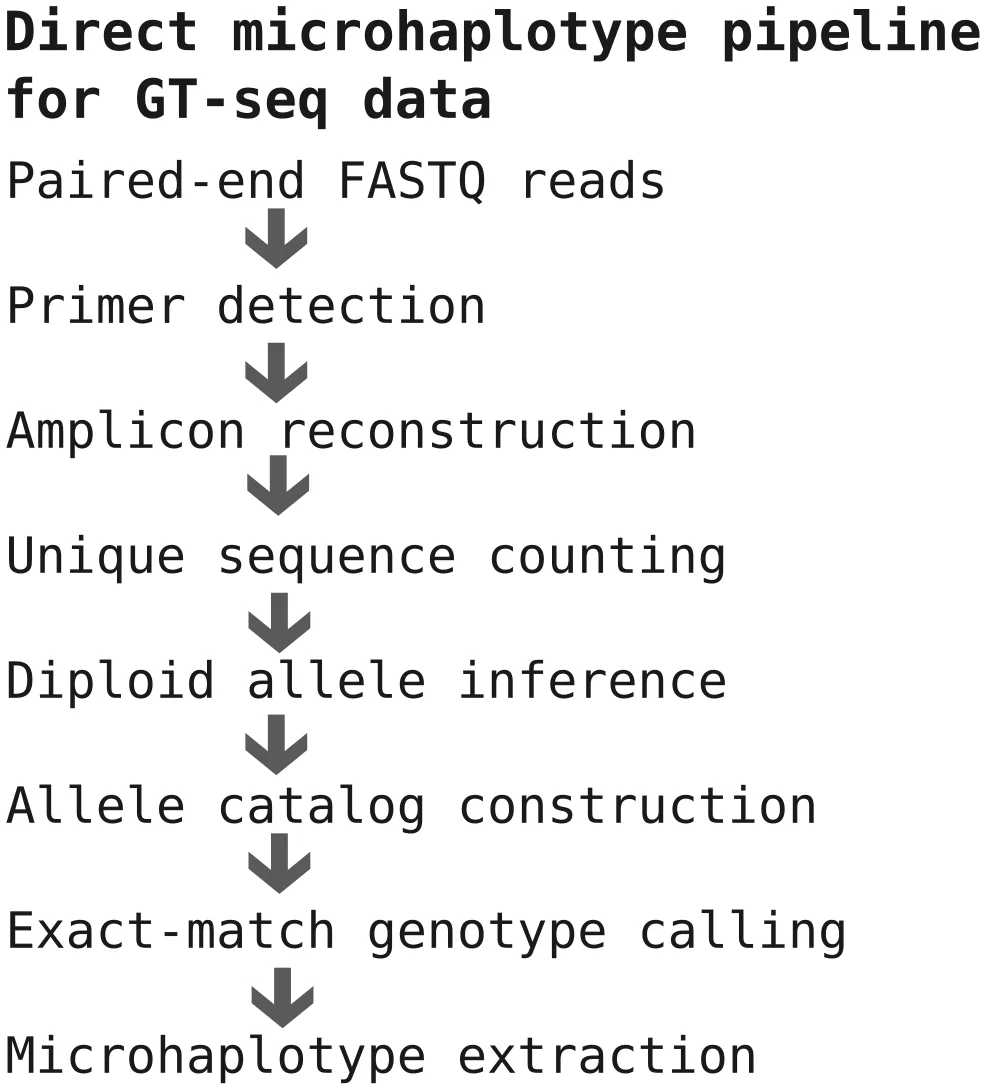
Direct microhaplotype genotyping pipeline for GT-seq amplicon sequencing data. Overview of the analytical workflow implemented in gtseq_microhap. Paired-end FASTQ reads are scanned to identify primer-bounded amplicons corresponding to targeted loci, and paired reads are resolved into quality-aware consensus amplicon sequences. In the first analytical pass, unique amplicon sequences are ranked by read abundance within each sample to identify present diploid alleles and construct a catalog of observed haplotypes for each locus. In the second pass, sequencing reads are assigned to catalog haplotypes by exact sequence matching, diploid genotypes are called using an abundance-based model, and phased microhaplotypes are extracted from catalog allele sequences.

The analysis described here is implemented as a single open-source Python script developed for processing paired-end amplicon sequencing data generated by GT-seq panels. The script accepts paired-end FASTQ files and a table of primer sequences defining the targeted loci. The software performs four primary steps: (1) resolution of paired-end reads into primer-bounded amplicon sequences, (2) identification of candidate allele sequences and construction of a locus-specific allele catalog, (3) genotype inference using an abundance-based diploid model, and (4) extraction of phased microhaplotype representations from catalog haplotypes. The implementation is designed to operate directly on high-depth amplicon sequencing reads without requiring read alignment to a reference or use other conventional variant-calling pipelines. The software is designed to operate on standard paired-end FASTQ files such as those produced by Illumina or Element sequencing platforms (Fig 1).

### GT-seq library preparation and sequencing

Genomic DNA samples from delta smelt (*Hypomesus transpacificus*) were genotyped using a GT-seq amplicon sequencing panel consisting of 410 targeted loci. The panel included loci selected from prior marker discovery efforts, including candidate microhaplotypes identified before panel development. GT-seq library preparation followed the general protocol described by Campbell et al. [1], in which locus-specific primer pairs are multiplexed in a single PCR. However, the indexing and normalization steps were performed using Nate’s Plates normalization and tagging kits (GTseek LLC, Twin Falls, Idaho, USA). The pooled GT-seq library was sequenced using an Element AVITI sequencing instrument at the University of Minnesota Genomics Center (UMGC). Sequencing was performed using paired-end (PE100) chemistry, producing paired FASTQ files for each individual sample.

To evaluate cross-platform reproducibility, the same pooled GT-seq library was subsequently sequenced on an Illumina NovaSeq X instrument using paired-end short-read chemistry at SeqMatic (Fremont, CA). FASTQ files generated from the Illumina run were processed using the same analysis pipeline described above. For cross-platform comparison, genotype inference for the Illumina dataset was performed using the allele catalog constructed from the Element dataset, allowing direct comparison of genotype calls across sequencing platforms without re-generating locus-specific catalogs. This approach ensures that any differences observed reflect differences in sequencing data rather than differences in allele discovery or catalog construction.

### Example dataset

To demonstrate the performance of the direct microhaplotype pipeline, a subset of sequencing data from this GT-seq panel was used as an example dataset. The dataset consists of paired-end (PE100) FASTQ files from 96 delta smelt (*Hypomesus transpacificus*) individuals prepared with the 410-locus GT-seq panel. Delta smelt is a diploid species, making it appropriate for evaluation under the diploid abundance model implemented here. To reduce file size while preserving locus representation and genotype structure, sequencing reads were subsampled to 150,000 read pairs per individual. The resulting dataset provides sufficient sequencing depth to demonstrate allele discovery, catalog construction, and genotype inference using the direct microhaplotype pipeline.

The example dataset is publicly available at Zenodo: https://doi.org/10.5281/zenodo.19069550. The Zenodo archive contains the example FASTQ dataset, primer definition file, reference-derived validation VCF, validation scripts, reproducibility documentation, and all supplementary analyses presented in this study.

### Primer-bounded paired-end read resolution

GT-seq libraries consist of PCR amplicons generated from locus-specific primer pairs and sequenced using paired-end short-read platforms. Because each locus is defined by known primer sequences, these primers provide natural anchors for identifying and reconstructing the targeted amplicon sequences.

**Fig 2:**
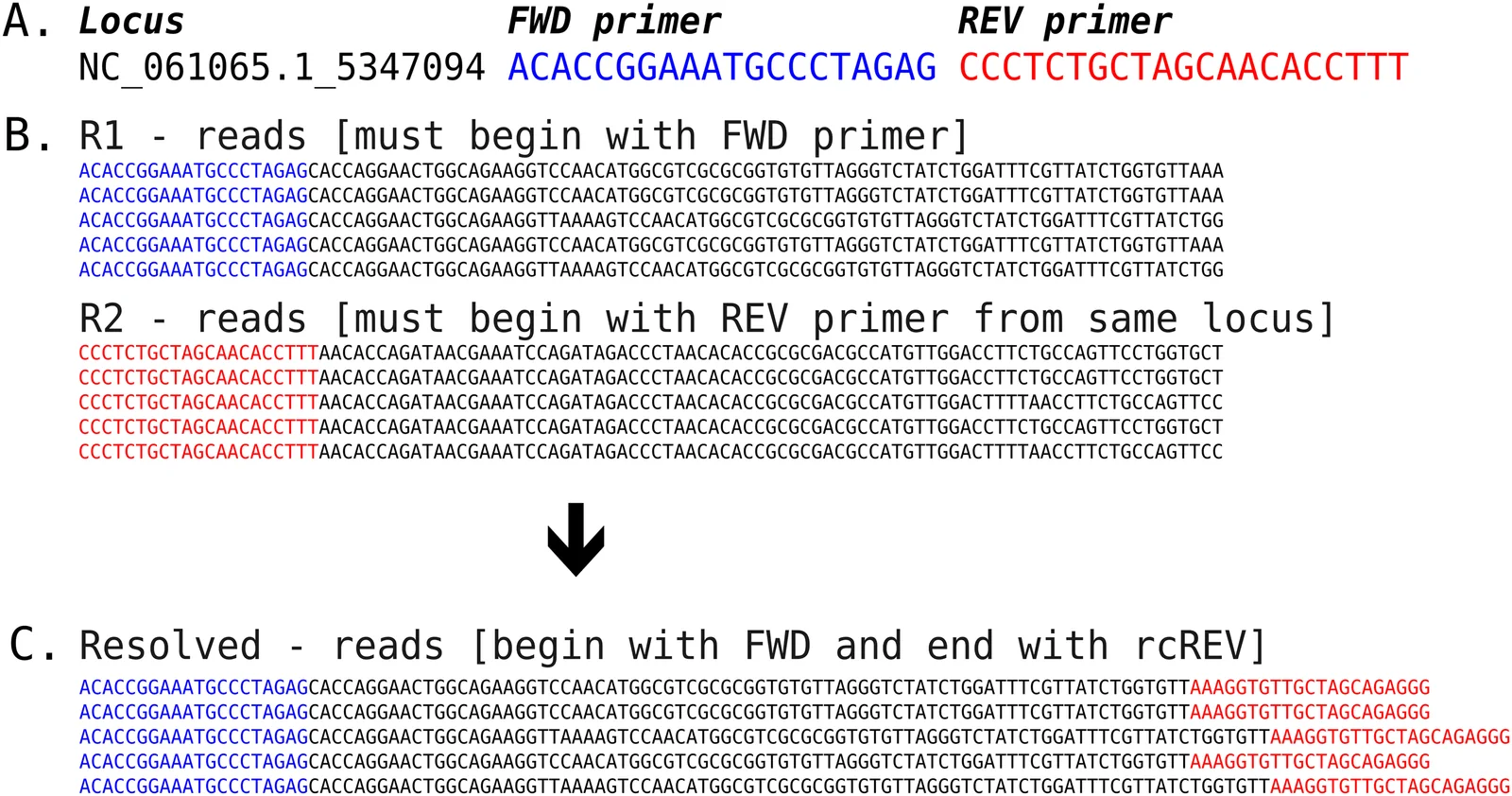
Resolution of primer-bounded amplicon sequences from paired-end reads. **(A)** GT-seq paired-end sequencing reads contain locus-specific forward and reverse primer sequences that define the boundaries of each amplicon. Read pairs are retained only when the expected forward and reverse primer combination is detected for the same locus. **(B)** Validated paired-end reads are resolved into complete primer-bounded consensus amplicon sequences using quality-aware read-pair reconstruction. Resolved sequences are normalized to a common orientation beginning with the forward primer and ending with the reverse complement of the reverse primer, providing the input for downstream allele discovery and genotype calling.

The analysis pipeline scans paired-end FASTQ files to identify reads beginning with a forward primer sequence corresponding to a defined locus. Candidate reads are then evaluated using their paired read to confirm the presence of the corresponding reverse primer sequence. Only read pairs containing the expected primer combination are retained for downstream analysis (Fig 2A).

For validated read pairs, the complete primer-bounded amplicon sequence is reconstructed using both paired-end reads. Read pairs are first oriented relative to the primer sequences and then resolved into a single consensus amplicon using per-base quality scores. When the full amplicon is contained within Read 1, Read 2 provides independent verification of overlapping bases and resolves sequencing discrepancies in favor of the higher-quality nucleotide. For longer amplicons, sequence extending beyond Read 1 is recovered from Read 2, allowing complete reconstruction of the primer-bounded amplicon. This quality-aware read-pair resolution generates a single high-confidence consensus sequence for each amplicon prior to allele discovery and genotype calling while reducing the impact of sequencing errors among observed amplicons (Fig 2B).

All reconstructed sequences are normalized to a single orientation beginning with the forward primer and ending with the reverse complement of the reverse primer. The resulting primer-bounded amplicon sequences are written to locus-specific FASTQ files and represent the fundamental unit of analysis for subsequent allele discovery and genotyping.

### Allele discovery and catalog construction

Allele discovery is performed using the primer-bounded amplicon sequences obtained from the read resolution stage. For each sample and locus, unique amplicon sequences are identified and their read counts tabulated. Because GT-seq data typically exhibit very high read depth per locus and Illumina and Element sequencers produce high-quality reads with low error rates, true allelic sequences occur at substantially higher frequencies than sequencing errors. Within each sample and locus, unique sequences are therefore ranked by read abundance. Under a diploid model, at most two alleles are expected per locus in each individual. Accordingly, the two most abundant sequences are retained as candidate alleles. Additional filtering is applied based on allele abundance ratios. For example, when the most abundant sequence constitutes the vast majority of reads at a locus, the locus is classified as homozygous and only the dominant sequence is retained as the candidate allele. When two sequences occur at substantial frequencies, both are retained as candidate alleles representing a heterozygous genotype. Once all candidate alleles have been identified across all samples, the sequences are aggregated to construct a catalog of unique haplotypes for each locus. Each distinct amplicon sequence is assigned a unique allele identifier. The resulting catalog represents the set of observed haplotypes across the dataset and serves as the reference for genotype calling in the second pass of the analysis.

### Genotype inference using a diploid abundance model

Following construction of the allele catalog, the primer-bounded amplicon sequences are reanalyzed in a second pass to assign reads to catalog haplotypes. Each resolved sequence is compared against the allele catalog for the corresponding locus using exact sequence matching. Exact sequence matching minimizes erroneous reassignment among closely related haplotypes, while the abundance-based allele discovery stage removes the vast majority of sequencing artifacts before genotype inference.

For each sample and locus, reads matching each catalog allele are counted. The two alleles with the highest read counts are retained as observed alleles for that sample. Genotype classification is based on the proportion of reads corresponding to the second most abundant allele (A2). Samples with a second-allele proportion below a defined threshold (default = 10%) are classified as homozygous, whereas samples with substantial representation of two alleles (25% – 50%) are classified as heterozygous. The A2 metric is defined as zero when only a single catalog allele is observed. Thresholds are configurable, with default values determined empirically from high-depth GT-seq datasets. Loci with insufficient read depth or ambiguous allele ratios are classified as low confidence or no-call. This abundance-based approach leverages the high sequencing depth typical of GT-seq assays to distinguish true allelic variation from sequencing noise.

### Microhaplotype extraction and phased SNP representation

The allele catalog generated in the previous step consists of complete amplicon sequences representing observed haplotypes for each locus. Catalog haplotypes are positionally compared to identify polymorphic sites, allowing phased SNP and indel variation to be extracted. Because each allele sequence represents a contiguous DNA fragment observed in sequencing reads, the relative phase of polymorphisms within the locus is inherently preserved. Consequently, the resulting haplotypes represent phased combinations of SNPs and indels within each amplicon.

For each locus, the polymorphic sites are extracted from the catalog haplotypes to generate phased SNP representations of each allele. These phased haplotypes constitute microhaplotypes, defined as short genomic regions containing multiple linked polymorphisms that can be observed within a single sequencing read or reconstructed amplicon.

Microhaplotype genotypes are therefore represented as combinations of phased allele sequences rather than individual unlinked SNP calls. This representation captures the joint information from multiple polymorphic sites within each amplicon and can substantially increase the information content of loci used for kinship inference, parentage analysis, and population genetic applications.

### Software implementation

The analysis workflow described above is implemented in the Python script gtseq_microhap_catalog_and_call.py, available through the GTseq GitHub repository: https://github.com/GTseq/gtseq_microhap

The software accepts paired-end FASTQ files and a table of locus-specific primer sequences as input and performs read resolution, allele catalog construction, genotype inference, and microhaplotype extraction in a single pipeline. The implementation is designed to operate directly on high-depth targeted amplicon sequencing data generated by GT-seq assays without requiring reference alignment or conventional variant-calling workflows.

### Independent validation of direct microhaplotype genotype calls

To evaluate the accuracy of genotype calls produced by the direct microhaplotype pipeline, we implemented an independent reference-based validation workflow using the same primer-resolved amplicon sequences generated by the pipeline.

Beginning with the resolved FASTQ files produced during the primer-bounded read reconstruction stage, each sample was aligned independently to the *Hypomesus transpacificus* reference genome using BWA-MEM. Primer-resolved reads were used rather than the original paired-end FASTQ files because they represent complete reconstructed amplicons with primer sequences retained and sequencing adapters removed, thereby eliminating complications associated with read-through adapter sequence and paired-end overlap while ensuring that both analytical methods operated on identical biological observations.

The resulting alignments were coordinate sorted and indexed using SAMtools. Variant discovery was then performed using the standard BCFtools mpileup/call workflow. The resulting VCF was filtered to retain high-quality biallelic SNPs suitable for direct comparison with variants emitted by the direct microhaplotype pipeline.

Version 0.3.1 of the direct microhaplotype pipeline includes an optional VCF export mode (--emit-vcf) that projects phased catalog haplotypes onto genomic coordinates using BWA alignment of catalog allele sequences. Catalog haplotypes are aligned to the reference genome to determine genomic position and reference/alternate alleles for each polymorphic site while retaining genotype calls made by the abundance-based diploid model. The resulting VCF therefore represents SNP genotypes derived from direct microhaplotype inference while remaining fully compatible with standard VCF-based analysis software.

Genotype concordance was evaluated by matching variants between the two VCF files using chromosome, genomic position, reference allele, alternate allele, and sample identity. Concordance statistics were calculated across all shared non-missing genotype calls. Missing genotype categories were tracked separately to distinguish differences attributable to conservative genotype filtering from true genotype discordance.

### Genotype accuracy across sequencing depth

To evaluate the robustness of abundance-based genotype inference at reduced sequencing depth, primer-resolved FASTQ files were randomly downsampled on a per-locus basis to maximum depths of 10, 25, 50, 100, and 200 reads per locus. Downsampled datasets were genotyped independently using the same exact-match catalog assignment algorithm and abundance-based diploid genotype model used for the full dataset. The minimum read depth required for genotype calling was maintained at the default threshold of 10 assigned reads per locus. Genotypes called from each downsampled dataset were compared with those generated from the complete dataset to calculate (1) genotype concordance among loci successfully genotyped at both depths and (2) genotype call recovery, defined as the proportion of full-depth genotype calls successfully recovered after downsampling.

### Marker information content

To quantify the information gain obtained by treating GT-seq amplicons as microhaplotypes rather than single SNPs, marker-level polymorphism information content (PIC) was calculated for both microhaplotype genotypes and single-SNP genotypes derived from the same sequencing data. For each microhaplotype locus, the corresponding single-SNP genotype was selected using the polymorphic site with the highest minor allele frequency within the locus. PIC, expected heterozygosity, allele number, and genotype call rate were calculated independently for each marker using observed allele frequencies. Marker-level statistics were compared across all loci represented in both datasets.

## Results

### Primer-bounded read reconstruction efficiently recovered GT-seq amplicons

The full delta smelt dataset consisted of 96 individuals prepared using a 410-locus GT-seq panel. Across all samples, the sequencing run produced 234,935,633 raw read pairs, corresponding to an average of 2,447,246 raw read pairs per sample. Application of the primer-bounded read resolution step retained 164,209,863 primer-bounded read pairs, corresponding to an average of 1,710,519 primer-bounded read pairs per sample. Overall, 69.9% of raw read pairs were retained as primer-bounded reads suitable for downstream allele catalog construction and genotype inference. Among individual samples, raw read counts ranged from 1,255,265 to 3,270,346 read pairs, while primer-bounded read counts ranged from 166,594 to 2,602,617 read pairs. Most samples exhibited consistently high recovery of primer-bounded reads, although a small number of lower-performing samples were observed.

### Direct microhaplotype inference from reconstructed amplicons

To illustrate the method, locus NC_061065.1_5347094 was examined in detail. This locus contained multiple polymorphic positions, including both SNPs and a short indel variant. Because the allele sequences span the full amplicon, the phase of these linked polymorphisms was preserved directly in the catalog haplotypes, allowing generation of phased SNP genotypes.

**Fig 3:**
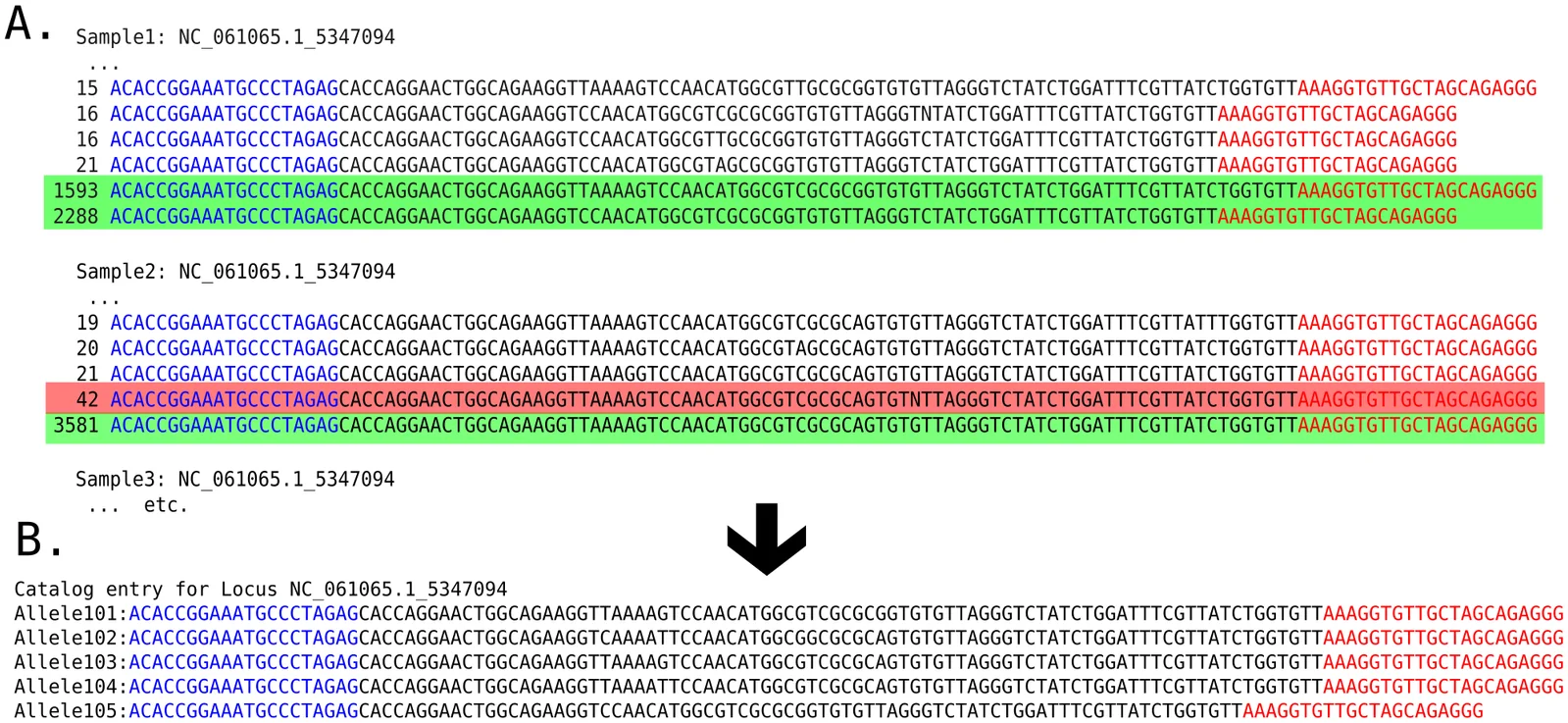
Diploid abundance model for allele discovery and catalog construction. **(A)** Primer-bounded amplicon sequences from each sample are collapsed into unique sequences and ranked according to read abundance. Under the diploid abundance model, the top one or two most abundant sequences are retained as observed haplotype alleles, while low-frequency sequences are treated as sequencing artifacts and excluded from catalog construction. **(B)** Candidate alleles identified across all samples are aggregated to construct a catalog of unique haplotypes for each locus. Each unique haplotype is assigned an allele identifier that serves as the reference for subsequent genotype calling.

**Fig 4:**
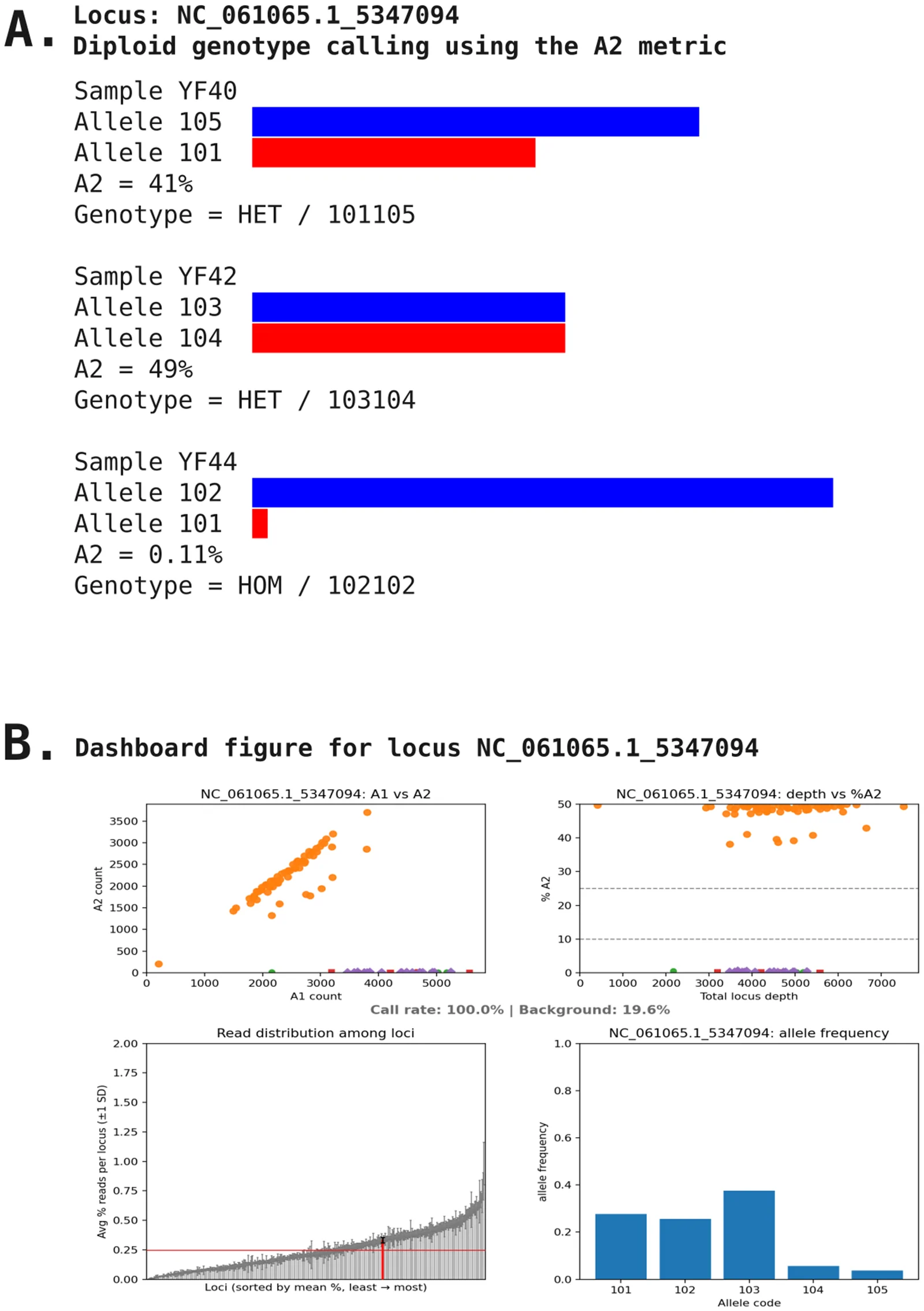
Diploid genotype calling using the A2 abundance metric. **(A)** Reads matching catalog haplotypes are counted for each sample and locus. Genotypes are called by the relative abundance of the second most common allele (A2), with heterozygous samples exhibiting two abundant alleles and homozygous samples exhibiting a single dominant allele. **(B)** Locus-level quality dashboard summarizing genotype performance for an example locus, including allele abundance, A2 values, read depth distribution, and allele frequency. These summaries facilitate visualization of genotype quality and locus performance across all samples.

Following read resolution (Fig 2), primer-bounded sequences were grouped by locus and unique amplicon sequences were ranked by read abundance within each sample. These candidate alleles were aggregated across samples to construct a catalog of observed haplotypes for each locus (Fig 3). In the second pass of the resolved FASTQ files, primer-bounded reads were assigned to catalog haplotypes by exact sequence matching, allowing diploid genotype inference based on allele abundance (Fig 4A).

**Fig 5:**
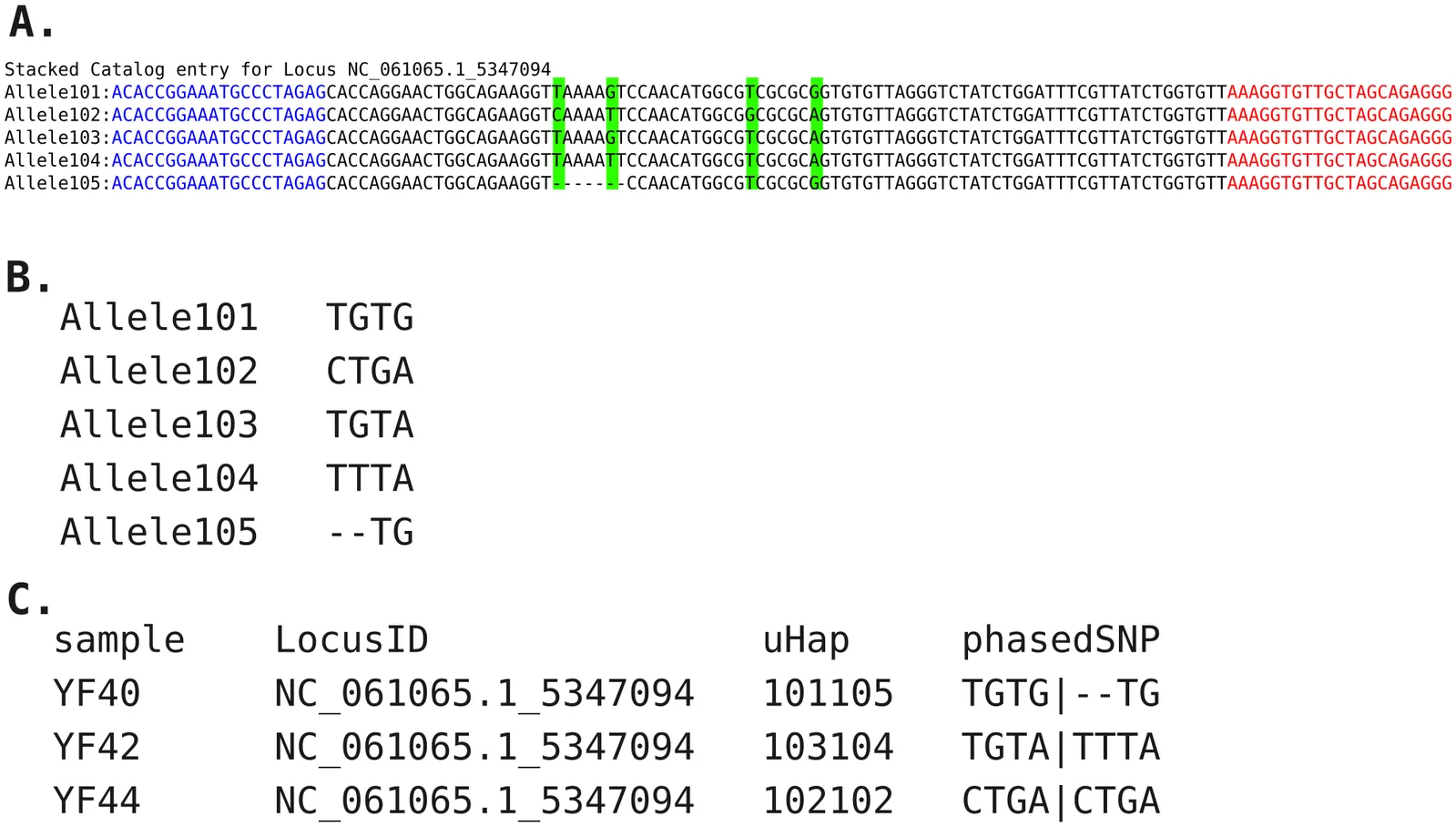
Extraction of phased microhaplotypes from catalog haplotypes. **(A)** Catalog haplotypes are aligned to identify polymorphic positions within the reconstructed amplicon, including both single nucleotide polymorphisms and insertion–deletion variants. **(B)** Variable positions are extracted from each catalog allele to generate phased microhaplotype representations while preserving the linkage phase observed in the original sequencing reads. **(C)** Diploid genotypes are represented as combinations of phased haplotype alleles, producing multi-allelic microhaplotypes suitable for downstream analyses including population genetics, parentage assignment, and kinship assignment.

This haplotype-first framework recovered phased haplotypes directly from GT-seq amplicon sequences without requiring read alignment or conventional variant calling (Fig 1). Because GT-seq amplicons typically span the full region containing the targeted polymorphisms, haplotype sequences could be reconstructed directly from sequencing reads following exact-match genotyping (Figs 1 and 5).

### Abundance-based diploid genotype calling

Diploid genotypes were called from allele-specific read counts using the relative abundance of the second most frequent allele (A2). For each sample and locus, reads matching catalog haplotypes were counted and ranked by abundance. The two most abundant alleles were retained as the candidate diploid genotype, and the proportion of reads corresponding to the second-most abundant allele was used to distinguish heterozygous and homozygous genotypes.

Examples from locus NC_061065.1_5347094 illustrate this approach (Fig 4A). In heterozygous individuals, two haplotypes were observed at similar frequencies, typically with A2 proportions near 0.5. In contrast, homozygous individuals exhibited extremely low A2 values, reflecting the presence of only a single dominant haplotype at the locus. This abundance-based model provided a simple and robust method for genotype inference using high-depth amplicon sequencing data.

Across the dataset, locus-level genotype summaries revealed the distribution of haplotypes and diploid genotypes across samples (Fig 4B). These locus dashboards provide a compact representation of allele frequencies and genotype composition for each microhaplotype locus.

### Extraction of phased microhaplotypes

Catalog haplotypes were positionally compared within each locus to identify polymorphic sites, including both single nucleotide polymorphisms and short insertion–deletion variants (indels). Because each haplotype sequence represents a contiguous DNA fragment derived from a single primer-bounded amplicon, the relative phase of polymorphisms within the locus was preserved directly in the catalog haplotypes.

Locus NC_061065.1_5347094 contained multiple polymorphic positions that could be resolved into distinct haplotype sequences (Fig 5). These haplotypes represent phased combinations of SNPs and indels within the amplicon and therefore constitute microhaplotypes. Diploid genotypes at this locus were represented as pairs of haplotype sequences, producing phased microhaplotype genotypes suitable for downstream population genetic and kinship analyses.

Because haplotypes are reconstructed directly from sequencing reads rather than inferred from independent SNP calls, the resulting microhaplotypes retain the full linkage information contained within each amplicon. This representation captures the joint information from multiple polymorphic sites and can substantially increase the effective information content of loci compared to single SNP markers.

### Cross-platform reproducibility

To assess cross-platform reproducibility of the direct microhaplotype pipeline, genotype calls generated from the Element dataset were compared to those obtained from the Illumina NovaSeq X sequencing run. Across 410 loci and 96 individuals, genotype concordance between platforms was 99.96% for loci successfully genotyped in both datasets (S1 Fig). The majority of discrepancies were attributable to loci with low read depth or marginal genotype quality, resulting in no-call versus genotype comparisons rather than conflicting genotype assignments. True conflicting genotype assignments between platforms were rare relative to comparisons involving no-calls.

Sample-level concordance between corresponding Element and Illumina samples exceeded 99.5% for all individuals, with most samples exhibiting greater than 99.9% genotype agreement (S2 Fig). Pairwise comparisons across all samples confirmed that each individual’s highest similarity was observed with its corresponding sample across platforms, with no evidence of sample misassignment or systematic platform bias.

### Agreement with an independent alignment-based workflow

To independently evaluate the accuracy of the direct genotype inference algorithm, we compared VCF output generated by the newly implemented --emit-vcf functionality of the microhap pipeline with VCF output produced by a conventional reference-alignment workflow. Rather than comparing raw paired-end sequencing reads, both analytical approaches were supplied with the identical primer-resolved amplicon sequences generated during the first stage of the microhap pipeline. This design isolated the genotype inference algorithms while eliminating potential differences arising from primer detection, paired-end merging, or adapter trimming.

The primer-resolved FASTQ files were aligned to the Hypomesus transpacificus reference genome using BWA-MEM, followed by variant calling with the standard BCFtools mpileup/call workflow. Independently, the direct pipeline projected reconstructed catalog haplotypes onto the reference genome using the new --emit-vcf functionality to generate a standards-compliant VCF representing SNP genotypes called directly from phased microhaplotypes.

Comparison of the two VCF files identified 1,085 shared SNPs across 96 individuals, resulting in 102,520 comparable non-missing genotype calls. Of these, 102,178 genotypes were identical between methods, corresponding to an overall genotype concordance of 99.67% (S3 Fig). The haplotype-first pipeline conservatively withheld 1,636 genotype calls that were assigned by the alignment-based workflow, whereas no genotype calls were observed exclusively in the direct haplotype-first pipeline, indicating that the abundance-based genotype classifier preferentially avoided uncertain genotype assignments rather than generating unsupported calls.

Inspection of discordant genotypes demonstrated that disagreements were concentrated within a relatively small subset of loci and were overwhelmingly attributable to homozygous-versus-heterozygous classifications rather than complete genotype reversals (0/0 versus 1/1). Furthermore, because several discordant SNPs frequently occurred within the same microhaplotype locus, many apparent SNP-level disagreements reflected a single haplotype-level classification difference rather than multiple independent genotyping errors. Collectively, these results demonstrate that genotype calls produced by the direct haplotype reconstruction algorithm are highly consistent with those generated by an independent reference-alignment-based variant-calling workflow.

### Robust performance across sequencing depth

To evaluate the robustness of direct microhaplotype genotyping across a range of sequencing depths, primer-resolved reads were downsampled prior to exact-match genotype inference. Even at the minimum supported genotype threshold of 10 reads per locus, genotype concordance with the full-depth dataset remained 99.46% among successfully genotyped loci, although only 13.8% of full-depth genotype calls met the minimum depth requirement of 10 × exact-matched catalog sequences (S4 Fig). Increasing sequencing depth substantially improved genotype recovery while maintaining consistently high genotype accuracy.

At 25 reads per locus, call recovery increased to 97.2% with 99.67% genotype concordance. Recovery exceeded 98.8% at 50 reads per locus and remained above 99.1% at 100 and 200 reads per locus, while genotype concordance exceeded 99.8% at all depths of 50 reads per locus or greater. These results demonstrate that the abundance-based genotype classifier remains highly accurate even near the minimum calling threshold and that moderate sequencing depths are sufficient to recover nearly all genotype calls observed in the full dataset.

### Microhaplotypes increase marker informativeness

To quantify the information gained by analyzing complete microhaplotypes rather than individual SNPs, marker-level polymorphism information content (PIC) was compared between microhaplotype genotypes and the highest-minor-allele-frequency SNP derived from the same GT-seq amplicons. Across 410 matched loci, microhaplotypes exhibited substantially greater allelic diversity than single SNP markers, averaging 4.19 alleles per locus compared with the inherently biallelic nature of single SNPs (2.00 alleles per locus). This increase in allelic diversity translated into substantially higher marker information content.

Mean PIC increased from 0.358 for single SNPs to 0.510 for microhaplotypes, while median PIC increased from 0.366 to 0.528. Across 410 loci, treating GT-seq amplicons as microhaplotypes increased mean PIC by approximately 42%, demonstrating that substantial information is routinely discarded when these loci are reduced to single SNPs. These results demonstrate that reconstructing complete haplotypes preserves substantially more genetic information than analyzing individual SNPs from the same amplified regions (S1 Table).

## Discussion

### Direct microhaplotype inference from amplicon sequencing data

This study demonstrates that GT-seq data can be analyzed directly as phased microhaplotypes rather than collections of independent SNPs, allowing existing GT-seq panels to recover substantially more genetic information without modification to laboratory protocols. Conventional analytical pipelines typically genotype predefined variants within targeted amplicons, either by identifying allele-specific sequences using internal probe sequences or by mapping sequencing reads to a reference genome and calling variants at targeted loci [1,5]. While effective for many applications, these workflows do not fully exploit the structure of targeted amplicon data where complete haplotypes can often be reconstructed directly from sequencing reads.

Several analytical tools have been developed to extract microhaplotypes from massively parallel sequencing data. Many of these methods rely on alignment-based workflows in which sequencing reads are first mapped to a reference genome and haplotypes are subsequently reconstructed from combinations of SNP calls. For example, the MICROHAPLOT software package identifies microhaplotypes from aligned sequencing reads and provides tools for analyzing haplotype structure across targeted loci [9]. While these approaches are effective when reference genomes are available and read alignment is reliable, they treat haplotypes as a derived product of variant calls rather than the primary signal present in sequencing reads.

The framework described here takes an alternative approach by identifying haplotypes directly from sequencing reads prior to identifying SNP variation. Because GT-seq amplicons frequently span the full targeted region, haplotypes can be reconstructed directly from primer-bounded reads without reference alignment or statistical phasing. In this workflow, polymorphic sites are identified only after the haplotype catalog has been constructed. This inversion of the traditional analysis order—identifying haplotypes first and extracting SNP variation second—provides a simplified and computationally efficient strategy for microhaplotype genotyping from high-depth amplicon sequencing data.

Unlike conventional alignment-based pipelines, the direct framework also preserves complete phased haplotype sequences throughout genotype inference while optionally generating standards-compliant VCF output for compatibility with existing downstream analysis software. Consequently, users can analyze GT-seq data as native multi-allelic microhaplotypes while maintaining interoperability with established SNP-based analytical workflows.

### Diploid abundance model and catalog construction

Central to this workflow is the diploid abundance model used to infer candidate alleles and construct locus-specific haplotype catalogs. Because GT-seq assays typically generate very high read depth per locus and Illumina and Element sequencing platforms produce predominantly high-quality reads, true allelic sequences are expected to occur at substantially higher frequencies than sequencing artifacts. Within each sample and locus, unique amplicon sequences are therefore ranked by read abundance and the two most abundant sequences are retained as candidate diploid alleles.

Aggregating these candidate alleles across samples produces a catalog of unique haplotypes for each locus that can subsequently be used for genotype inference. This abundance-based strategy provides a simple and robust approach for distinguishing true allelic sequences from sequencing artifacts in high-depth amplicon datasets.

Catalog construction assumes that each locus represents a single genomic target in a diploid organism. When multiple genomic regions are amplified by the same primer pair—such as duplicated loci or non-specific amplification events—more than two high-frequency sequences may be observed within a single sample. Under these conditions the diploid abundance model cannot unambiguously determine which sequences represent the true alleles. Rather than attempting to infer genotypes under ambiguous conditions, the algorithm intentionally fails to produce genotype calls for such loci, resulting in empty output for the affected marker. In practice this behavior serves as a useful diagnostic indicator of problematic loci, highlighting primer sets that amplify multiple genomic targets or duplicated regions of the genome. Such loci are typically identified and excluded during panel design through in silico specificity screening and empirical optimization, as performed during development of the example dataset used here. However, application of this pipeline to existing GT-seq panels may result in missing data for some loci.

Because the current implementation assumes a maximum of two alleles per locus per individual, the method is most appropriate for single-copy loci in diploid organisms. Nevertheless, the underlying framework could be extended to accommodate more complex genotype models. For example, a modified implementation could retain the top four sequences within each sample and apply abundance-based genotype inference appropriate for tetraploid organisms.

### Validation of the direct analytical framework

Three independent validation analyses demonstrate the accuracy, reproducibility, and robustness of the direct microhaplotype framework.

First, genotype calls generated from the same GT-seq library sequenced independently on Element and Illumina platforms exhibited exceptionally high agreement. Cross-platform genotype concordance exceeded 99.9% for nearly all samples, with the small number of discrepancies consisting primarily of no-call versus genotype comparisons rather than conflicting genotype assignments (S1 and S2 Figs). These results demonstrate that direct microhaplotype inference is highly reproducible across modern short-read sequencing platforms.

Second, comparison against an independent BWA/BCFtools workflow demonstrated 99.67% genotype concordance across 102,520 genotype comparisons spanning 1,085 SNPs in 96 individuals (S3 Fig). Importantly, both analytical workflows operated on identical primer-resolved amplicon sequences, isolating differences in genotype inference rather than differences arising from read preprocessing or alignment. The remaining discordant genotypes were concentrated within a relatively small subset of loci and predominantly reflected homozygous-versus-heterozygous classifications rather than complete genotype reversals, suggesting that these differences primarily arise from alternative genotype-calling models and thresholding decisions rather than systematic errors in haplotype reconstruction. The asymmetry in missing genotype calls, in which the direct pipeline occasionally withheld genotype assignments while producing no unsupported genotype calls relative to the alignment-based workflow, further reflects the intentionally conservative design of the abundance-based genotype classifier.

Finally, downsampling analyses demonstrated that genotype inference remained highly robust across a broad range of sequencing depths (S4 Fig). Although conservative depth filtering decreases genotype recovery near the minimum calling threshold, concordance among successfully genotyped loci remained greater than 99.4% even at the minimum supported depth of 10 reads per locus and exceeded 99.8% at sequencing depths of 50 reads per locus or greater. These results indicate that the abundance-based genotype model preferentially withholds uncertain genotype calls rather than producing incorrect genotypes, an important characteristic for applications requiring high-confidence genotype assignments.

Collectively, these independent validation analyses demonstrate that the direct microhaplotype framework is computationally accurate, reproducible across sequencing platforms, and robust across sequencing depths typical of GT-seq experiments.

### Microhaplotypes as highly informative genetic markers

Microhaplotypes have emerged as powerful genetic markers because they capture multiple linked polymorphisms within a single short genomic region. Unlike individual SNP markers, which are inherently bi-allelic, microhaplotypes produce multi-allelic loci with substantially higher heterozygosity and information content [6,7,10].

Because microhaplotypes represent phased combinations of SNPs and indels observed on the same DNA fragment, each locus can contribute considerably more information to relatedness analyses than a single SNP marker. Comparative studies have demonstrated that microhaplotype panels can outperform SNP panels when distinguishing closely related individuals or resolving complex pedigree relationships [8,11]. The multi-allelic nature of microhaplotypes increases their discriminatory power and reduces the number of loci required to achieve a given level of statistical confidence in kinship inference.

The present study provides empirical support for these theoretical advantages. Across the 410-locus validation panel, microhaplotypes exhibited higher polymorphism information content than the highest-minor-allele-frequency SNP at 364 loci, increasing mean PIC from 0.358 to 0.510 (S1 Table). This improvement reflects the additional genetic information retained when complete phased haplotypes are analyzed rather than reducing each amplicon to a single representative SNP.

### Implications and applications

The direct microhaplotype pipeline described here provides a practical framework for extracting phased microhaplotypes directly from GT-seq amplicon sequencing data. By operating directly on primer-bounded amplicon sequences, the method avoids the need for reference alignment during genotype inference while preserving complete haplotype structure. At the same time, optional reference-anchored VCF output maintains compatibility with existing SNP-based analytical software when required.

Because many ecological and conservation genomics studies already rely on GT-seq panels for genotyping large numbers of individuals, the ability to extract microhaplotypes from these datasets has the potential to substantially increase the information content of existing marker panels without requiring changes to laboratory protocols. Existing GT-seq assays that span multiple polymorphic sites can therefore be converted directly into multi-allelic microhaplotype marker systems using only analytical improvements.

More broadly, this work demonstrates how haplotype-centric analytical approaches can simplify the interpretation of targeted sequencing data. By treating complete haplotypes as the primary analytical signal and identifying individual polymorphisms only after haplotypes have been reconstructed, the direct microhaplotype framework provides an efficient, accurate, and reproducible strategy for genotyping high-depth amplicon sequencing data while preserving substantially more genetic information than conventional single-SNP analyses. Because the method operates entirely in software, many existing GT-seq datasets and published panels can immediately be reanalyzed as multi-allelic microhaplotypes without generating new laboratory data.

## Supporting information

S1 Fig Locus-level genotype concordance between Illumina NovaSeq X and Element AVITI sequencing platforms. Genotype calls generated independently from Illumina NovaSeq X and Element AVITI sequencing of the same 96 delta smelt individuals were compared across all 410 GT-seq loci. Each cell represents the proportion of matching genotype calls between platforms for a given locus. Concordance exceeded 99.9% for the vast majority of loci, demonstrating high reproducibility of direct microhaplotype genotyping across sequencing technologies.
(TIFF)

S2 Fig Pairwise sample-level genotype concordance between Illumina NovaSeq X and Element AVITI sequencing platforms. Heat map showing pairwise genotype concordance among all individuals sequenced independently on Illumina NovaSeq X and Element AVITI platforms. Each sample exhibited its highest concordance with the corresponding individual sequenced on the alternate platform, confirming correct sample identity and the absence of systematic sample misassignment.
(TIFF)

S3 Fig Genotype concordance between the direct microhaplotype pipeline and a conventional reference-based variant calling workflow. Genotypes generated by gtseq_microhap were compared with genotypes obtained by aligning primer-resolved amplicons to the *Hypomesus transpacificus* reference genome using BWA-MEM followed by variant calling with BCFtools. Concordance was evaluated across all shared biallelic SNPs identified by both approaches. Overall genotype concordance was 99.67% across 102,520 genotype comparisons, demonstrating close agreement between direct microhaplotype genotyping and a conventional alignment-based workflow.
(TIFF)

S4 Fig Effect of sequencing depth on genotype recovery and concordance. Primer-resolved reads were randomly subsampled over a range of sequencing depths to evaluate the effect of read depth on genotype recovery and accuracy. Genotype call rate and genotype concordance relative to the full-depth dataset were calculated independently at each subsampling level. Results demonstrate that genotype recovery decreases progressively with reduced sequencing depth, whereas genotype concordance remains high among successfully called genotypes across a broad range of sequencing depths.
(TIFF)

S1 Table Comparison of marker informativeness between highest-minor-allele-frequency SNPs and direct microhaplotypes. Marker summary statistics calculated from 410 loci in the delta smelt validation dataset. Single-SNP statistics were calculated using the highest-minor-allele-frequency SNP identified within each amplicon. Direct microhaplotype statistics were calculated from complete phased amplicon haplotypes reconstructed by gtseq_microhap. Microhaplotypes exhibited greater polymorphic information content (PIC), expected heterozygosity, and numbers of observed alleles per locus than the corresponding single-SNP representation.
(PDF)
