# Supplementary material for: Direct microhaplotype genotyping for GT-seq (Genotyping-in-Thousands by Sequencing) using a diploid abundance model
Source: PLoS Comput Biol. 2026 Sep 18;22(9):e1014808. doi: 10.1371/journal.pcbi.1014808 (PMC13607797; doi:10.1371/journal.pcbi.1014808)
Supplement: Supplementary file 1 [file pcbi.1014808.s005.pdf]

Supplementary Table S1. Comparison of marker informativeness between highest-minor-allele-frequency SNPs and direct microhaplotypes.

Marker summary statistics calculated from 410 loci in the Delta Smelt validation dataset. Single-SNP values were calculated using the highest-minor-allele-frequency SNP identified within each amplicon. Microhaplotype values were calculated from complete phased amplicon haplotypes reconstructed by gtseq_microhap.

Metric	Highest-MAF SNP	Direct microhaplotype
Number of loci evaluated	410	410
Mean polymorphic information content (PIC)	0.358	0.510
Median polymorphic information content (PIC)	0.366	0.528
Mean expected heterozygosity (He)	0.468	0.584
Median expected heterozygosity (He)	0.482	0.596
Mean number of alleles per locus	2.00	4.19
Median number of alleles per locus	2	4
